# Enhanced Nerve Regeneration by Bionic Conductive Nerve Scaffold Under Electrical Stimulation

**DOI:** 10.3389/fnins.2022.810676

**Published:** 2022-04-27

**Authors:** Zhenhui Liu, Yanshi Liu, Maimaiaili Yushan, Aihemaitijiang Yusufu

**Affiliations:** ^1^Department of Orthopedics, Henan Provincial People’s Hospital, Zhengzhou, China; ^2^People’s Hospital of Zhengzhou University, Zhengzhou, China; ^3^People’s Hospital of Henan University, Zhengzhou, China; ^4^Department of Trauma and Micro Reconstructive Surgery, The First Affiliated Hospital of Xinjiang Medical University, Urumqi, China

**Keywords:** PLLA fibrous mats, bionic conductive nerve scaffold, electrical stimulation, nerve repair, nerve regeneration

## Abstract

Repair of peripheral nerve defect (PND) with a poor prognosis is hard to deal with. Neural conduit applied to nerve defect at present could not achieve the effect of autologous nerve transplantation. We prepared bionic conductive neural scaffolds to provide a new strategy for the treatment of PNDs. The highly aligned poly (L-lactic acid) (PLLA) fiber mats and the multi-microchannel conductive scaffolds were combined into bionic conductive nerve scaffolds, which were implanted into rats with sciatic nerve defects. The experimental animals were divided into the scaffold group (S), scaffold with electrical stimulation (ES) group (S&E), and autologous nerve transplantation group (AT). The regenerative effect of bionic conductive nerve scaffolds was analyzed. Compared with aligned PLLA fiber mats (APFMs), highly aligned fiber mats had a higher fiber orientation and did not change the tensile strength, Young’s modulus, degradation rate, elongation at break of the fiber membrane, and biocompatibility. The bionic conductive nerve scaffolds were well matched with the rat sciatic nerve. The evaluations of the sciatic nerve in Group S&E were close to those in Group AT and better than those in Group S. Immunohistochemical results showed that the expression levels of neurofilament heavy polypeptide (NF-H) and protein S100-B (S100-β) in Group S&E were higher than those in Group S, and the expression levels of low-density lipoprotein receptor-related protein 4 (LRP4), mitogen-activated protein kinase (MAPK) p38, extracellular signal-regulated kinase (ERK), and mitogen-activated protein kinase kinase (MEK) in Group AT were higher than those in Group S. Bionic conductive nerve scaffolds combined with ES could enhance peripheral nerve regeneration and achieve satisfactory nerve regeneration close to autologous nerve grafts. ERK, p38 MAPK, MEK, and LRP4 may be involved in peripheral nerve regeneration under ES.

## Introduction

Peripheral nerve injury often leads to significant functional impairment and permanent disability. Despite modern diagnostic procedures and advanced microsurgical techniques, functional recovery after peripheral nerve repair was often unsatisfactory (Modrak et al., 2020). Autologous nerve transplantation (AT) was the gold standard for the repair of peripheral nerve defects (PNDs). However, AT could cause secondary trauma, neuroma formation, and permanent loss of sensation in the donor site. There were other problems such as limited supplied area, microstructural difference, and mismatch between donor and recipient nerves (Wu et al., 2016). Moreover, the results of AT were far from satisfactory, as not all patients could regain acceptable muscle strength and sensory function (Cheah et al., 2017). It was urgent to find better strategies for the repair of PND.

At present, the developed nerve conduit or scaffold for PND had been applied in clinical practice and achieved certain clinical effects. The nerve conduits developed for PND, including NeuraGen, NeuraWrap, NeuroMatrix/Neuroflex, Neurolac, and Neurotube, had been applied in clinical practice and achieved certain clinical effects (Chrzaszcz et al., 2018; Alike et al., 2019; Kornfeld et al., 2019; Rbia et al., 2019). Neural scaffolds prepared by electrospun silk fibroin were used to bridge the 30-mm sciatic nerve gap in dogs (Xue et al., 2018). A small gap sleeve showed a protective effect in bridging peripheral nerve mutilation (Yu et al., 2009). Composite biodegradable nerve conduit, mimicking peripheral nerve microenvironment, promoted the sciatic nerve regeneration and suppressed the oxidative stress (Qiu et al., 2014). Electrospun nanofibers, with interconnected porous microstructure, were a promising solution for PND. The microstructure provided by electrospun nanofibers, imitating the natural extracellular matrix, was useful for cell migration, adhesion, and proliferation (Xie et al., 2010). The oriented electrospun fibers were shown to guide regenerating axons to linear conformations and support axon growth (Kaplan et al., 2020). Biodegradable poly (L-lactic acid) (PLLA) had been extensively used for PND due to its biodegradability, porous structure for vascularization, and consistency in design requirements (Evans et al., 2002; Niu et al., 2021). Although some achievements had been made in the repair of PND with nerve conduit, significant functional recovery of nerve conduit is limited, as regenerating axons frequently grew long distances to reach denervated target organs, and the rate of regeneration was slow. Besides, regenerating axons were not confined to their original sheaths, which results in failure to reach their target end-organs (Scholz et al., 2009). Peripheral nerve had a natural multi-channel structure with the outer membrane, fascicular membrane, nerve fibers, connective tissues, blood vessels, and perineuria, which was a super three-dimensional structure with many functions, such as electrical conduction function.

**FIGURE 1:**
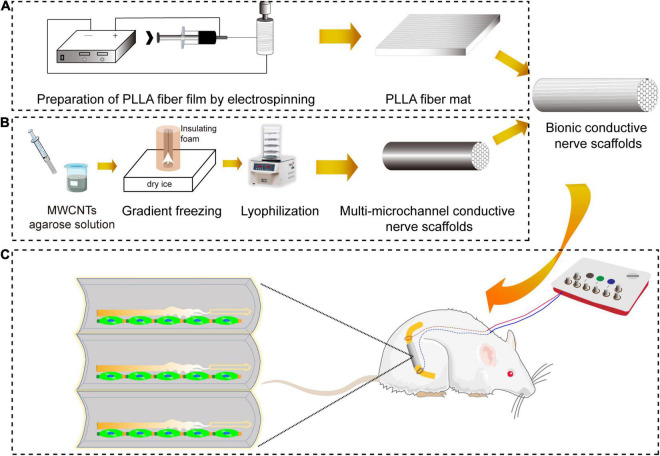
The schematic diagram of repair of sciatic nerve defects by bionic conductive nerve scaffold in rats. **(A)** The preparation of PLLA fiber mats by electrostatic spinning. **(B)** The preparation of customized conductive nerve scaffolds with multi-microchannels. **(C)** Nerve regeneration promoted by bionic conductive nerve scaffold combining ES *in vivo*. PLLA, poly (L-lactic acid); ES, electrical stimulation.

Electrical stimulation (ES) was found to have the function of promoting the speed and accuracy of motor axonal regeneration and sensory neuron regeneration to accelerate functional peripheral nerve regeneration (Al-Majed et al., 2000; Geremia et al., 2007; Haastert-Talini et al., 2011; Gordon, 2016). The preparation of the scaffold with electrical conductivity was key to the implementation of ES. Electroactive carbon nanotubes (CNTs) had attracted a lot of research interest (Corletto and Shapter, 2020). We had prepared electroconductive multi-microchannel scaffolds embedded in multiwall CNTs (MWCNTs) for neuron growth under ES (Liu et al., 2020). Based on our previous research, we prepared bionic conductive nerve scaffolds to combine with ES to obtain functional peripheral nerve regeneration and provide a new strategy for the treatment of PND, which is shown in Figure 1. The bionic conductive nerve scaffold had multiple microchannels, which simulated the internal nerve bundles of peripheral nerves and guided the growth of the nerve depending on the spatial structure to reduce the mismatch. Under the protection and guidance of PLLA fiber mats, conductive nerve scaffold combined with ES was used to speed neuron regeneration. Besides, we preliminarily discussed the molecules that may be involved in peripheral nerve regeneration under ES in this study.

## Materials and Methods

### Fabrication of Poly (L-Lactic Acid) Fiber Mats

Electrospun PLLA (120 kDa, Sigma, St. Louis, MO, United States) fiber mats were prepared by electrospinning from 13% PLLA solutions composed of methylene dichloride (DCM) and dimethylformamide (DMF). The parameters were set as the flow rate of spinning fluid of 1 ml/h, direct voltage of 24 kV, receiving distance of 15 cm, and continuous spinning time of 12 h. The diameter of the roller in the receiving device was 10 cm. The aligned PLLA fiber mats (APFMs) were fabricated with a rotating speed of the roller at 2,500 rpm. The highly APFMs (HAPFMs) were obtained with a rotating speed of 3,500 rpm.

### Characterization of Poly (L-Lactic Acid) Fiber Mats

APFMs and HAPFMs were sprayed with gold for 10 s and then observed by a scanning electron microscope (SEM; Hitachi, Tokyo, Japan, SU8010). The diameter and fast Fourier transform spectrogram (FFT) of fibers in SEM images were measured and analyzed by ImageJ software (Morrill et al., 2016).

Tests of the tensile mechanical strength of APFMs and HAPFMs cut into rectangular samples with a size of 40 mm × 10 mm were carried out on the mechanical testing machine (Bose Endura-Tec ELF 3200, NY, United States). The tensile stress–strain curves were drawn in which the tensile strength, Young’s modulus, and elongation at break were calculated.

The biodegradability of APFMs and HAPFMs was measured *in vitro*. The fiber mats to be tested were added to an EP tube with phosphate-buffered saline (PBS) and incubated in an incubator at 37°C and 5% CO_2_. The samples were removed, washed with distilled water, and dried on days 7, 14, 21, 28, 35, and 42. The degradation rate was calculated by the weight loss rate using the following formula.


$$\mathrm{Weight}\,\mathrm{loss}\,\mathrm{rate}=\frac{\mathrm{W0}-\mathrm{W1}}{\mathrm{W0}}\times 100\%$$

where W_0_ and W_1_ are the mass of the fiber mats before and after degradation, respectively.

The RSC96 cells (a spontaneously transformed rat Schwann cell (SC) line derived from the long-term culture of rat primary SC) (ZhouQiaoXinZhou, Shanghai, China, ZQ0154) were cultured in Dulbecco’s modified Eagle medium (DMEM) (Gibco, Grand Island, NY, United States) supplemented with 10% fetal bovine serum (FBS; Gibco), 100 μg/ml of streptomycin, and 100 U/ml of penicillin (BI) under a humidified atmosphere with 5% CO_2_ at 37°C. The RSC96 cells, seeded into the 96-well plates at a density of 2,000 per well, were cocultured with pre-wet APFMs and HAPFMs, which were cut into the same size. The optical density (OD) values of cells after cultivation for 1, 3, and 5 days were measured by Thermo Fisher Scientific Multiskan GO Spectrophotometer (Waltham, MA, United States) using Cell Counting Kit-8 (CCK-8) kit (Dojindo, Kumamoto, Japan).

The cells were added to APFMs and HAPFMs at a concentration of 5,000 per mat to coculture for 3 days. The fiber mats with RSC96 cells were fixed by 2.5% glutaraldehyde for 1 h, dehydrated by graded *tert*-butyl alcohol, and imaged by SEM.

### Fabrication of Bionic Nerve Scaffolds

Customized multi-microchannel conductive nerve scaffolds were prepared to match the diameter of the rat sciatic nerve. According to the instructions, polydimethylsiloxane (PDMS) composed of components A and B was mixed and added to the syringe (5 ml, ShanDongWeoGao) with an excisional top. Then the 18-G needle with a 1.5-mm diameter was inserted into the center of the syringe. After the PDMS was incubated at 37°C for 24 h, the syringe and needle were removed to obtain customized PDMS mold.

**FIGURE 2:**
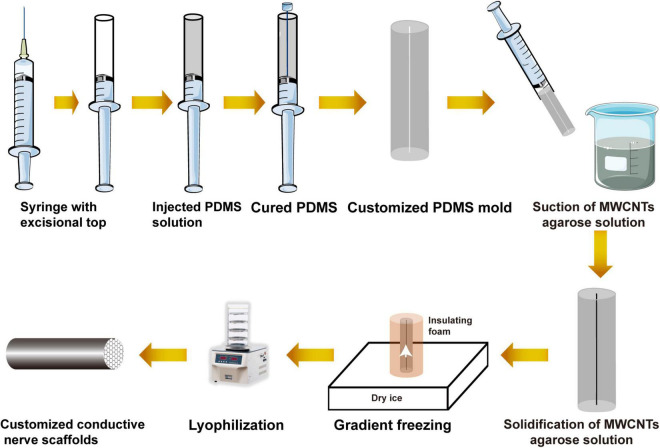
Schematic diagram of the preparation of the customized conductive nerve scaffold with multi-microchannel.

Multi-microchannel conductive nerve scaffolds were prepared and characterized in our previous study (Liu et al., 2020). Based on our previous results, MWCNT-agarose solution contained 3% wt agarose (Biowest, MAD, Spain) and 0.025% wt MWCNT (XFNANO Materials Technology, Nanjing, China). Fully dissolved MWCNT-agarose solution by heat was suctioned into the PDMS mold with a syringe. After having cooled and solidified, MWCNT-agarose gel and the mold were placed into insulating styrofoam except for their bottom, which was placed on dry ice to create a uniaxial low-temperature gradient for 2 h (Liu et al., 2020). Lyophilized for 24 h, customized conductive nerve scaffolds with multi-microchannel could be removed from the mold. The process is shown in Figure 2. Under the condition that the direction of fibers was consistent with the vertical axis of the scaffold, the multi-microchannel conductive nerve scaffold was wrapped by HAPFMs to harvest the bionic nerve scaffolds, which were cut into 10-mm length and was sterilized by Zhao et al. (2020) Co for further use.

### Animals and Surgical Procedures

The experimental animals were adult female specific pathogen-free Sprague Dawley (SPF SD) rats (200–250 g). Feeding and experimental operation were carried out in accordance with the regulations of the animal laboratory of Xinjiang Medical University, and the experimental process was approved by the Ethics Committee of Xinjiang Medical University (license no. IACUC-20150225-11). A total of 45 rats were randomly divided into three groups: (1) autologous nerve transplantation group (Group AT), (2) nerve scaffold group (Group S), and (3) nerve scaffold and ES group (Group S&E).

The right sciatic nerve was selected as the experimental side in each rat. After the rats were anesthetized by intraperitoneal injection of 7% chloral hydrate at the dose of 0.5 ml/100 g, the sciatic nerve was exposed under a stereomicroscope, and 10 mm was excised from the middle segment. The excised nerve was turned 180° and sutured with 8-0 non-injurious suture in Group AT. Nerve defects were bridged with the bionic nerve scaffolds, the membranes of which were sutured to the epineurium on both sides of the defect in Group S and Group S&E. Coupled electrodes were embedded on normal nerve 1 mm away from the suture sites. The electrode wires connected to the electrode reached the back of the neck through the subcutaneous tunnel and were fixed on the skin. In Group S&E, rats were given ES of 20-Hz continuous pulses (1 V, 0.1 ms, 1 h/day) for 7 days (Al-Majed et al., 2000). Rats in Group AT and Group S were fed routinely without ES. After ES of Group S&E was completed, all the electrodes and wires were removed under anesthesia. After surgery, all the rats were routinely fed, and their limbs were observed carefully. Protective covers were fixed on the experimental foot, and the integrity of the covers was checked daily to avoid self-mutilation.

### Analysis of Sciatic Functional Index

The motor functional recovery was assessed by the rats’ hind paws recorded on the white paper. The sciatic functional index (SFI) was calculated by the following formula (Varejão et al., 2001):


$$S\,F\,I=-38.3\times \frac{E\,P\,L-N\,P\,L}{N\,P\,L}+109.5\times \frac{E\,T\,S-N\,T\,S}{N\,T\,S}+13.3\times \frac{E\,I\,T-N\,I\,T}{N\,I\,T}-8.8$$

where PL is the length of paw, TS is the distance between toes 1 and 5, and IT is the distance between toes 2 and 4. E represents the data of experimental sides, and N represents the data of normal sides.

### Analysis of Electrophysiology

At the 4th, 8th, and 12th weeks after implantation, electrophysiological tests by biological function experimental system (BL-420S) were performed on the rats. The stimulation electrode placed approximately 2 mm on the proximal side of the graft site conducted ES, the surgical sciatic nerve was exposed, and the receiving electrode inserted into the distal gastrocnemius muscle recorded compound muscle action potential (CMAP) and nerve conduction velocity (NCV).

### Muscle Weight and Histological Analysis

At the 8 and 12th weeks after implantation, the bilateral gastrocnemius muscles were completely removed from the medial and lateral femoral condyles to the calcaneal tubercle to calculate the atrophy degree. After being weighed, the muscles were fixed in 4% paraformaldehyde solution for 24 h for further use. The relative wet weight ratio of the gastrocnemius was calculated by the following formula: muscle relative wet weight ratio = muscle weight of experimental leg/muscle weight of the contralateral side. The cross-sectional areas of the middle gastrocnemius fixed by paraformaldehyde were stained with Masson’s trichrome and observed under a microscope (Leica, Wetzlar, Germany). Five regions were randomly selected for each section. The muscle fiber diameter and the proportion of collagen fiber positive area were measured by using Image-Pro Plus 6.0 software.

### Histological Analysis of Regenerated Nerve

Regenerated nerves were harvested at the 8th, 12th, and 24th weeks after implantation. Cross sections were fabricated from the middle site of the regenerated nerve. Semi-thin (4-μm) sections were stained with H&E, Luxol Fast Blue (LBF), Masson’s trichrome, and Toluidine Blue (TB). Ultrathin sections (50 nm) were stained with uranyl acetate and lead citrate and observed by transmission electron microscopy (TEM; H-600 Electron Microscope, Hitachi, Japan). ImageJ software was used to measure the axon diameter and myelin sheath thickness of the regenerated nerves on non-overlapping TEM images (Amniattalab and Mohammadi, 2017). Then, the *g*-ratios (the ratio of the inner axonal diameter to the total outer diameter of the same axon with myelin) were calculated from the perimeters measured on TEM images. Twenty-five (corresponding to at least 25% of the total nerve cross-sectional area) (Magnaghi et al., 2011) fields of each slice were randomly captured under TEM. Five fibers randomly chosen in each field were measured using the ImageJ software.

### Immunohistochemical and Immunofluorescence Assays of Regenerated Nerve

The samples of the regenerated nerve at the 12th week after implantation were stained by immunohistochemical methods. The primary antibodies against S100-β (1:400, Abcam, Cambridge, United Kingdom), NF-H (1:200, Abcam), LRP4 (1:400, Abcam), p38 MAPK (1:200, Abcam), extracellular signal-regulated kinase (ERK) (1:200, Abcam), and MEK (1:200, Abcam) were diluted following the instructions provided by manufacturers. The positive area percentage of slices was analyzed by ImageJ software and compared among the three groups.

The slices of regenerated nerves from the 12th week after implantation were stained by the immunofluorescence method. Incubated with primary antibodies against S100-β (1:400, Abcam) or NF-H (1:200, Abcam), slices were incubated with secondary antibody coupled with Alexa Flour 488 (Abcam). A confocal microscope was used to observe and record the immunofluorescence result.

### Statistical Analysis

The orientation of PLLA fiber and the dispersion of RSC96 cells on PLLA fiber were indexes of qualitative observation. The quantitative data were presented as mean ± standard error and analyzed using SPSS software, version 23.0 (IBM, Armonk, NY, United States) to perform statistical analysis (unpaired Student’s *t*-test with Welch’s correction). Comparisons were performed by one-way ANOVA with a least significant difference (LSD) *post hoc* comparison in three groups. *p* < 0.05 was considered as statistical significance. GraphPad Prism software version 8.02 was used to draw a diagram of statistical results.

## Results

### Characterization of Poly (L-Lactic Acid) Fiber Mats

PLLA fiber mats prepared by electrospinning were white (see Supplementary Figure 1). There was no apparent difference between APFMs and HAPFMs. Observed by SEM, the fibers had a smooth appearance, uniform diameter, and porous structure distributed in the whole fiber mats. However, the fibers in HAPFMs had higher orientation than those in APFMs (see Supplementary Figure 1). The fiber diameter of APFMs was 506.66 ± 183.77 nm, and that of the HAPFMs was 626 ± 170.53 nm. There was no statistical difference in the fiber diameter between APFMs and HAPFMs.

**FIGURE 3:**
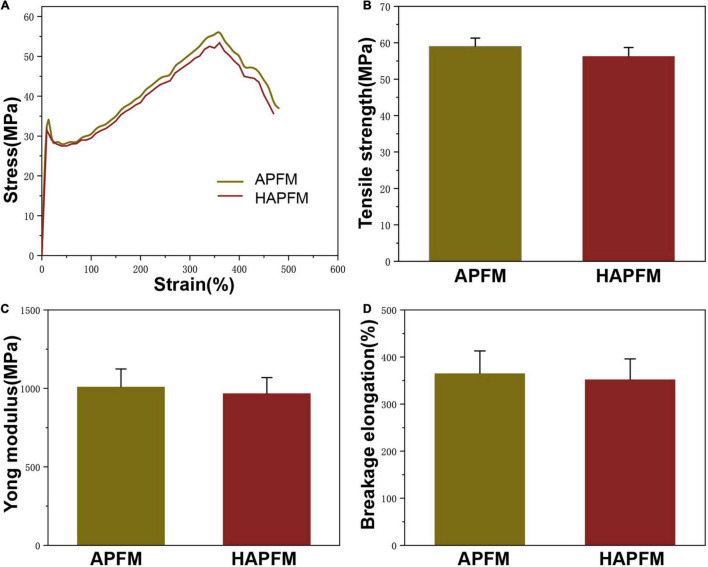
Mechanical properties. **(A)** Tensile stress–strain curve of APFMs and HAPFMs. **(B)** Tensile strength of APFMs and HAPFMs (*n* = 5, *p* > 0.05). **(C)** Young’s modulus of APFMs and HAPFMs (*n* = 5, *p* > 0.05). **(D)** Breakage elongation of APFMs and HAPFMs (*n* = 5, *p* > 0.05). APFMs, aligned poly (L-lactic acid) fiber mats; HAPFMs, highly aligned poly(L-lactic acid) fiber mats.

The mechanical properties were important parameters in tissue engineering applications. Figure 3A shows typical tensile stress–strain curves of APFMs and HAPFMs. According to this curve, the tensile strength, Young’s modulus, and breakage elongation of APFMs and HAPFMs were calculated. The tensile strength of APFMs was (59.06 ± 2.49) MPa, and that of HAPFMs was (56.82 ± 2.80) MPa. There was no significant difference in the tensile strength between the two types of fiber films (*p* > 0.05). Young’s modulus of APFMs was 1,037.07 ± 133.30 MPa, and that of HAPFMs was 971.45 ± 112.39 MPa. There was no significant difference in Young’s modulus between the two kinds of fiber membrane (*p* > 0.05). The breakage elongation of APFMs was 364.48 ± 53.79, and that of HAPFMs was 356.89 ± 40.21. There was no significant difference in the elongation at the break between the two types of fiber membrane (*p* > 0.05). Figure 3 shows that the increased orientation of PLLA fiber mats did not significantly change the tensile strength, Young’s modulus, and breakage elongation.

**FIGURE 4:**
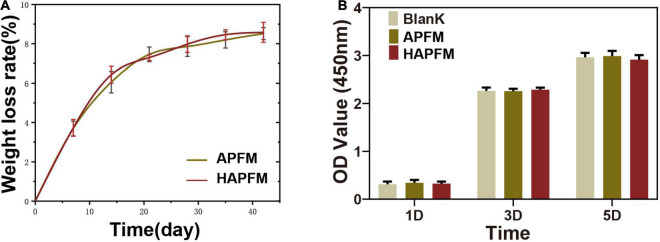
**(A)** Degradation rate curve of APFMs and HAPFMs. **(B)** OD value of RSC96 cells detected by CCK-8 method. There were no statistically significant differences in OD values in three groups at each time (*n* = 5, *p* > 0.05). APFMs, aligned poly (L-lactic acid) fiber mats; HAPFMs, highly aligned poly (L-lactic acid) fiber mats; OD, optical density; CCK-8, Cell Counting Kit-8.

The degradation rate of PLLA fiber mats was measured by simulating the physiological environment with PBS. Figure 4A shows that the change of the fiber orientation had no effect on the degradation rate of the PLLA fiber mats (*p* > 0.05). OD values, reflecting cell proliferation, were measured by the CCK-8 method, as shown in Figure 4B. There were no statistically significant differences in OD values in three groups at each time point (*p* > 0.05).

**FIGURE 5:**
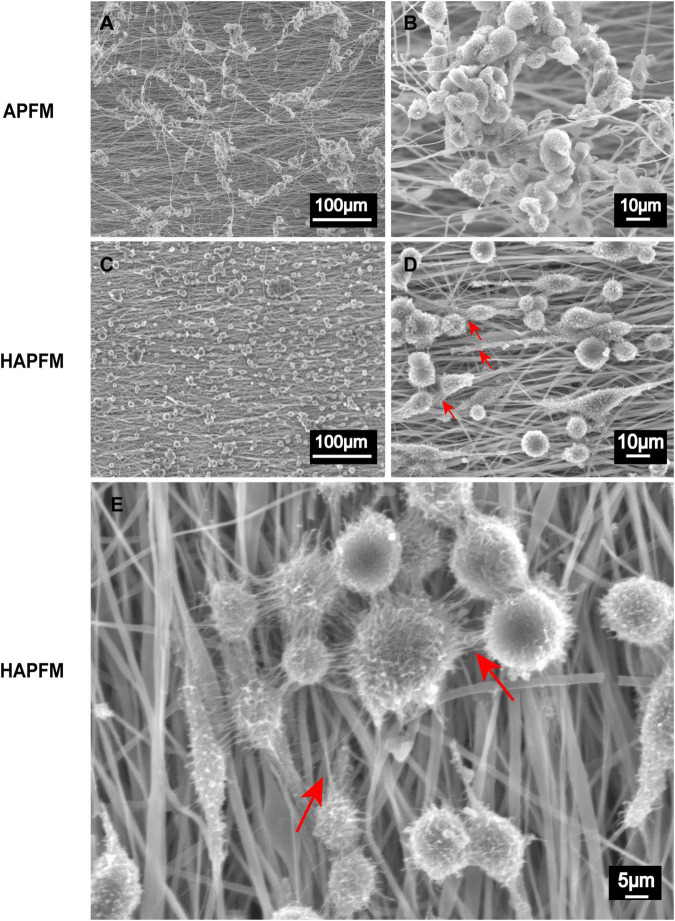
SEM images APFMs and HAPFMs with RSC96 cells. **(A,B)** The RSC96 cells growing on APFMs were distributed in clumps. **(C,D)** The RSC96 cells growing on HAPFMs showed relatively uniform distribution with filopodia (red arrow). **(E)** The filopodia (red arrow) of amplified RSC96 cells on aligned HAPFMs. SEM, scanning electron microscope; APFMs, aligned poly (L-lactic acid) fiber mats; HAPFMs, highly aligned poly (L-lactic acid) fiber mats.

RSC96 cells grew well and clustered on both APFMs and HAPFMs (Figure 5). However, cells dispersed and arranged along highly aligned fiber better on HAPFMs. Moreover, RSC96 cells on HAPFMs showed obvious filopodia, which not only played a role in the intercellular connection but also extended along the aligned fibers (Figure 5). But this phenomenon was not found on APFMs.

### Bionic Conductive Nerve Scaffolds

The prepared PDMS mold and the bionic conductive nerve scaffold are shown in Supplementary Figure 2. PDMS molds were transparent elastic cylinders with an inner diameter of 1.5 mm. Thus, the diameter of the customized conductive nerve scaffold by molds was 1.5 mm; HAPFMs and the customized conductive nerve scaffold were manufactured to the bionic conductive nerve scaffold with multi-microchannels.

### General Observation

Intraoperative conditions of rats are shown in Supplementary Figure 3. The electrodes and wires were firmly fixed to the skin. Inflammatory reactions such as redness and swelling were not observed around the electrodes and scaffolds. At the 12th week after implantation, the scaffold had degraded and nerve regenerated.

Gastrocnemius muscle atrophy of the operative side was observed in all groups. Muscle atrophy in Group S was more serious than that in the other groups. At the 12th week after surgery, gastrocnemius muscle atrophy was improved in all groups, with the best performance in Group AT and the worst in Group S.

### Results of Sciatic Functional Index and Electrophysiology

**FIGURE 6:**
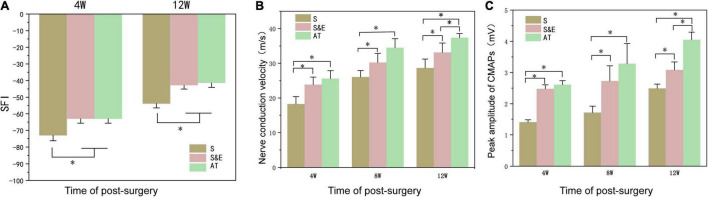
**(A)** At weeks 4 and 12, SFI values of Group S were lower than those of Group AT and Group S&E (*n* = 5, *p < 0.05). There was no significant difference in SFI values between Group AT and Group S&E. **(B,C)** The nerve conduction velocity of regenerated nerves and the peak amplitude of CMAP of the gastrocnemius muscle at the 4th, 8th, and 12th weeks after implantation (*n* = 5, *p < 0.05). SFI, sciatic functional index; CMAP, compound muscle action potential.

The motor function recovery of rats was evaluated by the analysis of SFI. SFI values ranged from 0 to −100, with 0 indicating normal function and −100 indicating a complete loss of function. Figure 6A shows the results of SFI in the three groups. At weeks 4 and 12, the SFI values of Group S were lower than those of Group AT and Group S&E (*p* < 0.05). However, Group AT and Group S&E had similar SFI values (*p* > 0.05).

Electrophysiological analysis was another tool to evaluate nerve regeneration. NCV and the CMAP peak amplitude of regenerated nerves at the 4th, 8th, and 12th weeks after implantation are shown in Figures 6B,C. At the 4th and 8th weeks postoperatively, the average amplitude and NCV of Group AT and Group S&E were higher than those of Group S, while there was no difference between Group AT and Group S&E (*p* > 0.05). In the 12th week, both mean NCV and mean NCV of Group S&E were higher than those of Group S, indicating that ES could promote nerve repair through nerve scaffold. The amplitude and NCV of Group AT were higher than those of Group S&E, suggesting that effects on nerve regeneration of ES weakened on the late stage of nerve repair *in vivo*. These results revealed that bionic nerve scaffolds combined with ES could achieve a similar regeneration effect to AT in the early stage of nerve repair.

### Results of Muscle Histology

**FIGURE 7:**
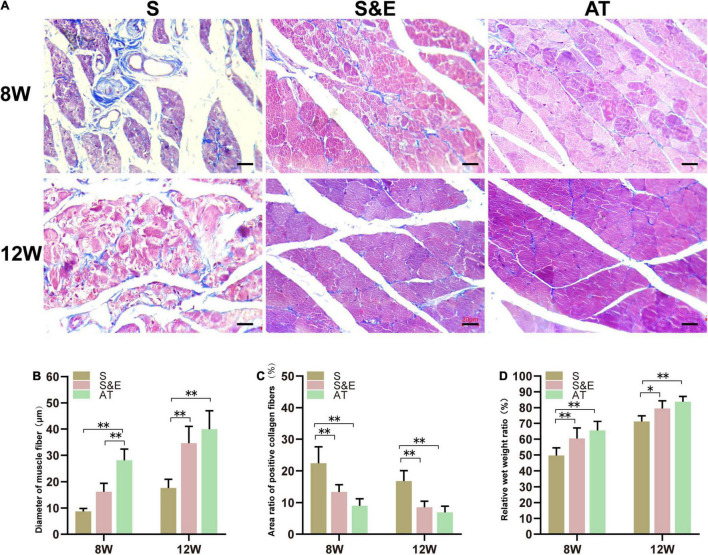
Results of muscle tissues at 8 and 12 weeks after implantation. **(A)** Masson’s trichrome staining of the gastrocnemius muscle on the experimental side of rats, scale = 20 μm. **(B)** Statistical results of muscle fiber diameter (*n* = 5). **(C)** Statistical results of the area ratio of positive collagen fibers (*n* = 5). **(D)** Statistical results of gastrocnemius relative muscle wet weight ratio (*n* = 5) (*p < 0.05, ^**^p < 0.01).

After sciatic nerve injury, the weight of the gastrocnemius muscle reduced due to atrophy of muscle fibers. As known, the muscles maintain their integrity due to a delicate balance between protein synthesis and protein degradation associated with equivalent rates of anabolic and catabolic processes. When the rate of protein degradation exceeds that of protein synthesis, the muscles can atrophy or waste away (Zizzo, 2021). The atrophied muscle could gradually recover with an increase of muscular reinnervation. At the time of sampling, it was generally observed that the gastrocnemius muscles on the experimental side were atrophic compared with those on the contralateral side. At the 8th week after implantation, the relative wet weight ratio in Group S was lower than that in both Group S&E and Group AT. At the 12th week after implantation, the relative wet weight ratio of all the groups reduced, but the ratio of Group S was still lower than that in both Group S&E and Group AT (Figure 7D).

The gastrocnemius muscles are atrophied due to the loss of the sciatic nerve, and the collagen fiber content in the muscle increases. Collagen fibers and muscle fibers in the gastrocnemius were distinguished by Masson’s trichrome staining. The muscle fibers were red, and the collagen fibers were blue. In Group AT, the muscle tissues were regular and full with a small number of collagen fibers distributed in the muscle space, while in Group S, the muscle fibers were atrophic, and the boundary was not clear. Moreover, plenty of blue collagen fibers scattered among the muscle bundles (Figure 7).

At the 8th week after implantation, the diameter of muscle fibers in Group AT was significantly higher than that in both Group S and Group S&E (*p* < 0.01). There was no statistical difference between Group S and Group S&E (*p* > 0.05). At the 12th week after implantation, the diameter of muscle fibers of all groups increased. However, Group S&E had a similar muscle fiber diameter to Group AT. The diameter of muscle fiber in Group S was significantly lower than that in both Group AT and Group S&E (*p* < 0.01). In order to quantify the collagen fiber ratio, the blue area ratio was statistically analyzed. At the 8th and 12th weeks, the collagen fiber ratio of Group S&E was similar to that of Group AT, while the collagen fiber ratio of Group S was higher than that of both Group S&E and Group AT (*p* > 0.05).

The results showed that Group S&E combined with ES achieved better nerve reinnervation of the repaired sciatic nerve than Group S.

### Histological Results of Regenerated Nerve

**FIGURE 8:**
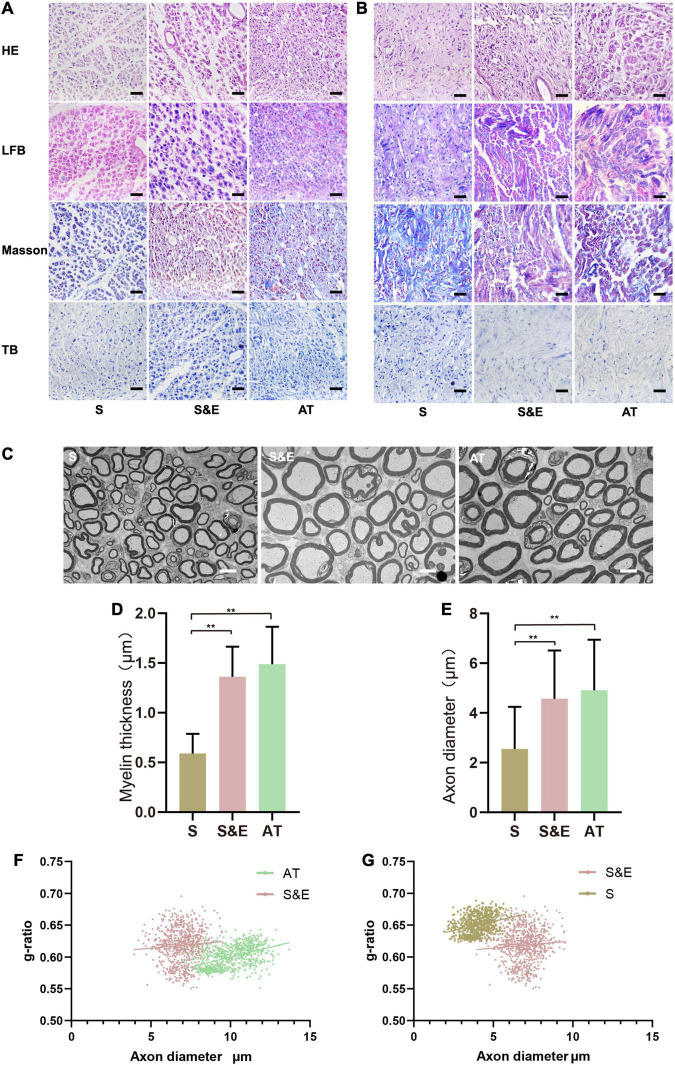
H&E, LBF, Masson’s trichrome, and TB staining images of the regenerated nerves. **(A)** The images at the 8th week after implantation. Scale = 25 μm. **(B)** The images at 12th week after implantation; scale = 25 μm. **(C)** The TEM images at 24th week after implantation; scale = 5 μm. **(D,E)** Statistical results of myelin sheath thickness and axonal diameter at 24th week after implantation (*n* = 5, **p < 0.01). **(F,G)** *g*-ratios of the regenerated nerve at 24th week after implantation. The *g*-ratio of Group AT was lower than that of Group S&E (*p* < 0.01), and the *g*-ratio of Group S&E was lower than that of Group S (*p* < 0.01). LBF, Luxol Fast Blue; TB, Toluidine Blue; TEM, transmission electron microscopy.

The slices of H&E staining showed that SC proliferated and migrated to the nerve defect to form new nerve tissues, and no obvious inflammatory reaction was observed. In the 8th and 12th weeks, it could be found that the number of regenerated cells in the AT group was larger and that the distribution of cells was more homogeneous than those of the other groups. However, H&E staining could not distinguish myelin sheath. LBF staining was used to observe the myelin sheath that was dyed blue. At the 8th week after implantation, the amount of myelin in Group S was less than that of the other groups, while plenty of myelin sheath in Group S&E and Group AT could be observed. At the 12th week after implantation, myelin sheath structures of the regenerated axons in all the groups could be seen clearly. However, intact myelin sheath was more easily observed in Group S&E and Group AT. Slices of Masson’s staining showed blue collagen fibers and red SC in the regenerative tissues. The collagen fiber content in Group S was higher than that of Group S&E and Group AT. Slices of TB staining could also show the regenerative axons and myelin sheath. At the 8th week after implantation, typical myelin structure could not be seen in cross sections of axons in all the groups. At the 12th week after implantation, the typical hollow myelin sheath structure of the blue ring could be observed in Group S&E and Group AT (Figures 8A,B). At the 24th week after implantation, regeneration of myelinated fibers was seen in all the groups (Figure 8C). However, the myelin sheath thickness of Group S was lower than that of both Group S&E and Group AT, while there was no statistical difference in myelin sheath thickness between the S&E group and the AT group. The axon diameter of Group S was lower than that of both Group S&E and Group AT, while there was no statistical difference in myelin sheath thickness between the S&E group and the AT group (Figures 8D,E). The *g*-ratio was widely utilized as a functional and structural index of optimal axonal myelination (Chomiak and Hu, 2009). Among the three groups, Group AT had the lowest *g*-ratio, while Group S had the highest *g*-ratio (Figures 8F,G). These results further indicated that ES could promote the growth of axon and myelination through a bionic nerve scaffold combined with ES.

### Immunohistochemical Results of Regenerated Nerve

**FIGURE 9:**
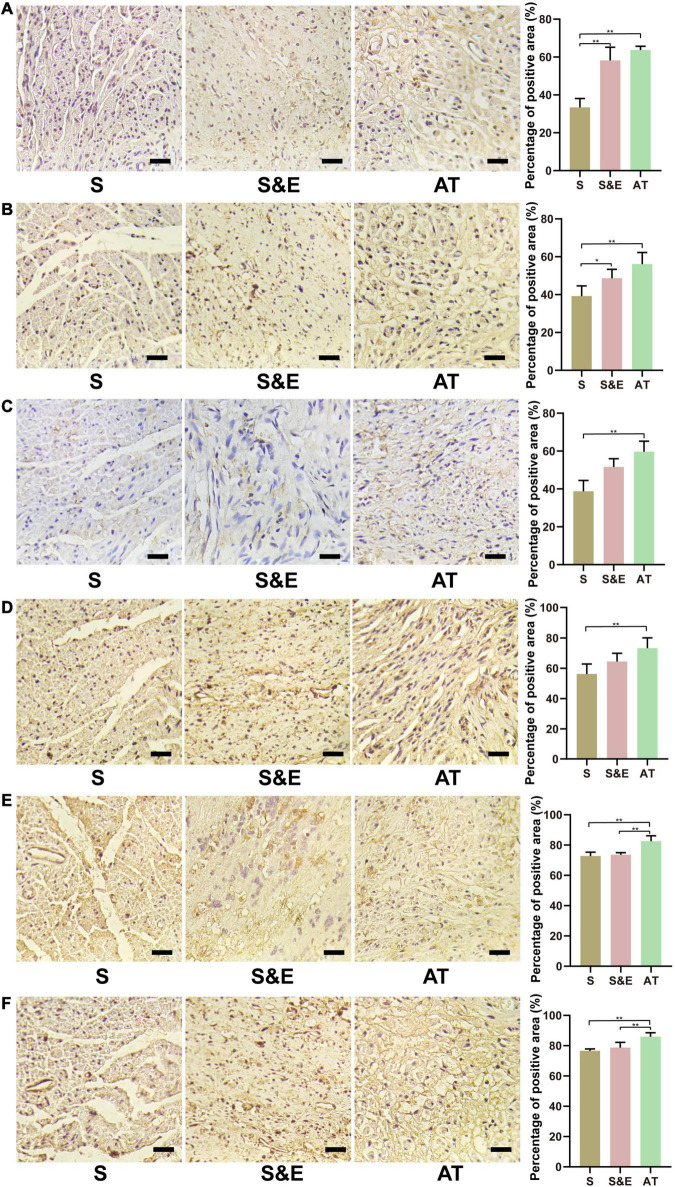
Immunohistochemical staining images and results at 12th week after implantation. **(A)** NF-H protein. **(B)** S100-β protein. **(C)** LRP4 protein. **(D)** p38 MAPK protein. **(E)** ERK protein. **(F)** MEK protein. Scale = 25 μm. *n* = 5, *p < 0.05, **p < 0.01.

Immunohistochemical methods were used to stain six proteins in the slices at the 12th week after implantation, including S100-β, LRP4, p38 MAPK, ERK, and MEK, to further evaluate the neural tissue regeneration and explore the possible mechanisms involved in nerve regeneration through protein expression (Figure 9). In the figure, samples of Group S were less compact than the other samples due to atrophy of the muscles. For treatment under the same conditions, all the samples were comparable.

The expressions of NF-H protein are shown in Figure 9A. The percentage of positive area in Group S&E and Group AT was higher than that in Group S (*p* < 0.01), while that in Group S&E was close to that in Group AT (*p* > 0.05). The expression differences of S100-β protein in each group were like NF-H, as shown in Figure 9B. Group S&E and Group AT were higher than Group S (*p* < 0.01), and the S&E group was close to the AT group, and there was no statistical significance between the two groups (*p* > 0.05).

The expressions of LRP4 protein are shown in Figure 9C. The percentage of positive area in Group AT was higher than that in Group S (*p* < 0.01), while there were no statistically significant differences between Group S and Group S&E, and between Group AT and Group S&E (*p* > 0.05). The expression differences of p38 MAPK protein in each group were like LRP4, as shown in Figure 9D. Group AT was higher than Group S. No difference was found between Group S and Group S&E, and between Group AT and Group S&E (*p* > 0.05).

The expressions of ERK protein are shown in Figure 9E. The area percentage of positive protein in Group AT was higher than that in Group S and Group S&E (*p* < 0.01).

There was no significant difference between the S&E group and the S group (*p* > 0.05). The expression differences of MEK protein in each group were like ERK, as shown in Figure 9F. Group AT was higher than Group S and Group S&E (*p* < 0.01). No difference was found between Group S and Group S&E (*p* > 0.05).

### Immunofluorescence Results of Regenerated Nerve

**FIGURE 10:**
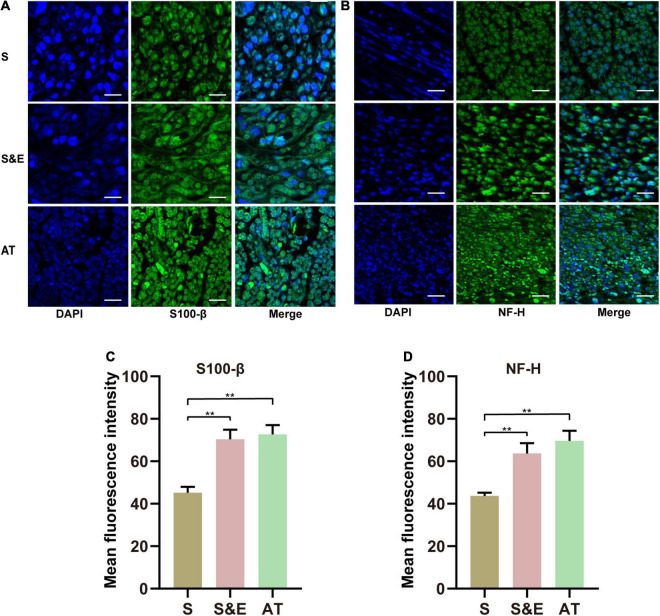
**(A,B)** S100-β and NF-H immunofluorescence images of regenerated nerves at 12th week after implantation. **(C,D)** Mean fluorescence intensity of S100-β and NF-H. Scales = 30 μm. *n* = 5, **p < 0.01.

At the 12th week after implantation, the regenerated nerve sections were immunofluorescence stained with NF-H and S100-β (Figures 10A,B). In those confocal cross-sectional images, NF-H and S100-β proteins are shown in green.

The statistical analysis of the average green fluorescence intensity in the images showed that the average NF-H fluorescence intensity in Group S was lower than that in Group S&E and Group AT (*p* < 0.01), while the average fluorescence intensity in Group S&E was close to that in Group AT (*p* > 0.05) (Figure 10C).

The fluorescence results of S100-β were like those of NH-H. The mean fluorescence intensity in Group S was lower than that in Group S&E and Group AT (*p* < 0.01), while the average fluorescence intensity in Group S&E was close to that in Group AT (*p* > 0.05) (Figure 10D).

## Discussion

We prepared a bionic conductive nerve scaffold constructed by HAPFMs and a conductive scaffold with multi-microchannels in this study. Combined with ES, the bionic conductive nerve scaffold implanted into the sciatic nerve defect of rats enhanced regeneration of the sciatic nerve and promoted myelination. Thus, motor function recovery was strengthened.

The conductive property of the nerve scaffold was endowed with MWCNT, which belonged to the CNT family. The CNTs were exploited for numerous applications in the biomedical field due to their vital electrical, chemical, thermal, and mechanical properties (Raphey et al., 2019). However, concerns related to their toxicity, biosafety, and biodegradation remained (Menezes et al., 2019). CNT dimensions, surface properties, bio-durability, and corona formation determined CNT toxicity (Lanone et al., 2013). Oxidative stress, membrane injury, and genotoxicity of CNTs were the major mechanisms behind the toxicity of CNTs (Saleemi et al., 2021). Nevertheless, any or a combination of these properties can be modified simultaneously for a given CNT, with a change in its overall toxicity, which could be extremely beneficial in the expansion of biomedical CNT applications (Lanone et al., 2013). Electrospun protein-CNT composite fibers induced polarization and activation of fibroblasts with cellular deficit (Chi and Wang, 2018). Collagen-CNT composite materials had successfully been used for tissue generation (MacDonald et al., 2005). In our previous research, agarose scaffolds with MWCNT appeared to have good biocompatibility (Liu et al., 2020). Thus, CNTs could be developed in many biomedical applications.

### Effect of Scaffold Structure on Peripheral Nerve Regeneration

Following AT, which was the gold standard for the repair of PND, the transplanted autologous nerve developed Wallerian degeneration and dissolved, leaving only the multi-channel frame structure without blood supply and conductive function. Thus, autologous nerves essentially provided a natural multichannel structure for nerve repair and failed to reproduce the microenvironment of nerve repair.

The bionic nerve scaffold prepared in this study contained multiple longitudinal microchannels through the scaffold, increasing the contact surface between cells and scaffold. In our previous research, we found that microchannels in the scaffold could provide the matrix for the attachment of SC (Liu et al., 2020). It was found that after being implanted in the sciatic nerve defect, multi-channel collagen scaffolds had reduced axon diffusion and promoted nerve function regeneration (Wieringa et al., 2018). However, although multichannel scaffolds could reduce axon dispersion, macroscopic channels did not provide enough physical clues for directional axon growth. Compared with multichannels, microchannels that provided topographic and physical signals could simulate better the multi-channel frame structure of the nerve. *In vivo*, collagen–chitosan scaffolds with longitudinal parallel microchannels had promoted nerve regeneration and functional recovery similar to those of autologous grafts when used to repair 15-mm sciatic nerve gaps in rats (Hu et al., 2009). This microstructure might affect SC migration and axon regeneration by interacting with cells.

The surface of scaffolds was also considered to be an important factor affecting cell attachment and proliferation (Moroder et al., 2011). MWCNTs in bionic conductive scaffolds increased the roughness of scaffolds to provide larger surface area and more complex micro-scale morphology, which was conducive to cell adhesion, growth, and the formation of cell–cell connections (Cho and Borgens, 2010; Saudi et al., 2019).

### Effect of Orientation Structure on Peripheral Nerve Regeneration

Compared with the random arrangement of fibers, the aligned fibers were more conducive to nerve cell differentiation and regeneration. Yang et al. (2005) found that the aligned fibers could promote the differentiation of neural stem cells. By staining the neuronal differentiation marker proteins, Mahairaki et al. (2011) found that neural stem cells on the aligned nanofibers had a higher rate of differentiation than those on the random fibers. Through qRT-PCR and immunohistochemical analysis, Cho et al. (2010) found that aligned nanofiber binding nerve growth factor (NGF) could effectively promote the differentiation of mesenchymal stem cells to neurons. The aligned fibers could guide the growth of dorsal root ganglion (DRG) neurites and promote the migration of astrocytes along the directional axis of the fibers, helping neurites pass through the injury site and regenerate in the peripheral nervous system (PNS) or spinal cord (Hurtado et al., 2011; Lin et al., 2018; Zhang et al., 2020).

PLLA had high clinical applicability due to its good biocompatibility, biodegradability, and thermoplastic processing (Kaplan et al., 2020). Approved by the Food and Drug Administration (FDA), PLLA scaffolds had been applied for clinical practice (Theodore et al., 2016). Various techniques could be used to process PLLA (Desimone et al., 2013; Gao et al., 2018). Electrospinning was a versatile and low-cost method to produce nanofibers. The electrospun fibers were similar to the protein fibers in the natural extracellular matrix and could provide appropriate contact guidance to promote neurite growth and nerve cell differentiation (Yu et al., 2015; Jahromi et al., 2020). Besides, the spatial structure of electrospun fibers was thought to facilitate the elongation of neurite (Wang et al., 2010).

With the development of electrospinning technology, higher aligned fibers had been prepared. However, it remained to be determined whether the use of higher aligned PLLA fibers could be more conducive to cell growth and application in peripheral nerve tissue engineering.

In this study, compared with APFMs, HAPFMs did not change the mechanical properties, degradability, and biocompatibility. However, we found that RSC96 cells on HAPFMs seemed to disperse more uniformly. Considering that the PLLA fiber mats could not provide strong support, we used the HAPFMs as the outer membrane of the bionic neural scaffold to simulate peripheral epineurium and provide physical protection for the neural scaffold.

### Effect of Electrical Stimulation on Peripheral Nerve Regeneration

ES played an important role in tissue regeneration. ES mediated wound healing in skin tissues, triggered cardiac pacing and rhythm in cardiac tissues, regulated bone homeostasis in bone tissues, and promoted nerve signal transmission in nerve cells (Ahadian et al., 2013; Thrivikraman et al., 2018). Thus, ES had been used as a bionic tool to regulate tissue behavior and regeneration. In animal models of peripheral nerve injury, ES could accelerate the growth of axons at the injured repair site and the re-myelination of regenerated axons, improve the innervation effect of target organs, and improve the regeneration of motor and sensory nerves (Zuo et al., 2020).

However, the molecular mechanism by which ES affects nerve regeneration was still unclear. It was suggested that ES simulated intracellular calcium ion waves at nerve dissociation sites, which traveled retrograde to the neuron cell body to produce a series of physiological effects (Love et al., 2018; McGregor and English, 2018). With the upregulated expression of neurotrophic factors [including brain-derived neurotrophic factor (BDNF)] elevated by ES, the content of cyclic adenosine monophosphate (cAMP) increased (Gordon, 2016; McGregor and English, 2018). Cytoskeleton assembly was enhanced by activating cAMP response element-binding protein (CREB), inhibiting Rho protein in the p75-Nogo receptor (p75-NgR) pathway, and upregulating Tα1 tubulin (Geremia et al., 2007; Gordon et al., 2010; Sit and Manser, 2011).

It was found that p38 MAPK may be involved in nerve regeneration. When p38 MAPK was inhabited, ES could not enhance neurite outgrowth *via* activation of CREB (Kawamura and Kano, 2019). Zhao et al. (2020) found that ES activated p38 MAPK and MAPK signaling pathways in regenerated nerves through conductive nerve scaffolds, thus enhancing axon growth after peripheral nerve injury. In our study, compared with Group AT, Group S had a lower expression of p38 MAPK. Therefore, assisted by ES, the expression of p38 MAPK in Group S&E was close to that in Group AT, which indicated that p38 MAPK played an important role in peripheral nerve regeneration. Protein kinases in the MAPK signaling pathway were crucial regulatory elements that controlled a variety of important cytological activities. These serine/threonine MAPKs transmitted extracellular signals into the cell to influence intracellular signal expression, thus promoting axon growth and myelination (Cargnello and Roux, 2011).

SC might be the main target cells of ES. SC activated by ES could release a variety of neurotrophic growth factors, including insulin growth factor-1, vascular endothelial growth factor (VEGF), hepatocyte-derived growth factor, NGF, BDNF, and heparin-binding protein-pluripotent growth factor (PTN) (Gordon et al., 2011). Neurotrophic factors bound to specific tyrosine receptor kinase (TRK) and p75 receptors activated corresponding signaling pathways to enhance peripheral nerve regeneration (Gordon, 2009). The TRK receptor signaling pathway acted through ERK1/2 (Romero et al., 2007). However, the expressions of both ERK and MEK in Group AT were higher than those in Group S&E and Group S in our research. Although Group S&E tended to be higher than Group S, the two groups did not show significant differences, which did not support the promotion of neural regeneration by ES through ERK or MEK pathways.

In the PNS, myelin sheaths from glial cells acted as insulators and sped up the transmission of signals from neurons, which was one of the important functions of SC. Peripheral nerve regeneration consisted of lengthening of axons and formation of the myelin sheath. As the main component of axons, NF-H was used to detect the effect of nerve regeneration. Our results showed that the expression of NF-H in Group S&E was higher than that in Group S, which suggested that ES could enhance the expression of NF in axons to facilitate axon regeneration. S100-β, a calcium-binding protein that was mainly found in glial cells, was often used to identify SC in the PNS. Immunofluorescence and immunohistochemical staining of S100-β in our study showed that the expression in Group S&E was higher than that in Group S, which suggested that ES could also increase the expression of S100β in SC. In brief, our results suggested that the application of ES combined with bionic conductive nerve scaffolds promoted neurite growth and myelin formation.

Besides, we observed that the expression of LRP4 in Group AT was higher than that in Group S. Thus, LPR4 might be involved in peripheral nerve regeneration. As one of the Agrin receptors expressed in skeletal muscle cells, LRP4 belonged to the low-density lipoprotein receptor family. In a zebrafish model of nerve regeneration, axons in wild-type zebrafish were regenerated properly, while the follower axons in LRP4 mutants could not regenerate through the injury gap, resulting in poor regeneration (Gribble et al., 2018).

LRP4 was widely recognized because it was involved in the Agrin/MuSK signaling pathway required for neuromuscular synaptic development (Dechiara et al., 1996). However, LRP4 may promote peripheral nerve regeneration through a pathway independent of Agrin/MuSK and the downstream effect factor Rapsyn. Compared with the wild type, zebrafish with mutational Agrin had developed a severe neuromuscular disorder, while peripheral nerve regeneration was not affected in these Agrin mutants (Gribble et al., 2018), since MuSK was an obligate signaling receptor for LRP4 during synaptic development and there was no defect of peripheral nerve regeneration in Rapsyn mutants. Thus, the signaling pathway through which LRP4 promoted nerve regeneration was still unclear.

Although expression of LRP4 in Group S&E had an incremental trend, we found no difference between Group S&E and Group S. We only treated Group S&E with ES for 1 week, so the physiological effects of ES on LRP4 may disappear at the 11th week after the ES had stopped. Even so, Group S&E achieved satisfactory nerve regeneration close to that of Group AT, indicating the importance of ES in the early stages of peripheral nerve regeneration.

### Limitations

There were some limitations in our study. First, the RSC96 cell line was used to evaluate the biocompatibility of PLLA fiber mats. Compared with SC, RSC96 cells expressed distinct proteins with potential biological significance (Ji et al., 2012). Second, although many researchers chose 12 weeks or less as endpoints (Gordon and Borschel, 2017; Chang et al., 2018; Rao et al., 2020), longer endpoints may be needed to evaluate the regenerative properties. Last, for the complexity of molecular mechanisms, we preliminarily discussed the possible molecules involved in peripheral nerve regeneration under ES in this study. However, ERK, p38 MAPK, MEK, and LRP4 and their phosphorylated forms at different times needed to be investigated in future studies.

## Conclusion

The bionic conductive artificial nerve scaffold was constructed by combining HAPFMs with the multi-microchannel conductive scaffold. After being implanted into the sciatic nerve defect of rats, bionic conductive nerve scaffolds combined with ES could enhance peripheral nerve regeneration and achieve satisfactory nerve regeneration close to autologous nerve grafts. ES may promote peripheral nerve regeneration through the MAPK signaling pathway. ERK, p38 MAPK, MEK, and LRP4 may be involved in peripheral nerve regeneration.

## Data Availability Statement

The raw data supporting the conclusions of this article will be made available by the authors, without undue reservation.

## Ethics Statement

The animal study was reviewed and approved by the Ethics Committee of First Affiliated Hospital of Xinjiang Medical University.

## Author Contributions

All authors listed have made a substantial, direct, and intellectual contribution to the work, and approved it for publication.

## Conflict of Interest

The authors declare that the research was conducted in the absence of any commercial or financial relationships that could be construed as a potential conflict of interest.

## Publisher’s Note

All claims expressed in this article are solely those of the authors and do not necessarily represent those of their affiliated organizations, or those of the publisher, the editors and the reviewers. Any product that may be evaluated in this article, or claim that may be made by its manufacturer, is not guaranteed or endorsed by the publisher.

## Abbreviations

PND: peripheral nerve defect
PLLA: poly (L-lactic acid)
S: scaffold
S&E: scaffold with electrical stimulation
AT: autologous nerve transplantation
NF-H: neurofilament heavy polypeptide
S100-B: protein S100-B
LRP4: low-density lipoprotein receptor-related protein 4
MAPK: mitogen-activated protein kinase
ERK: extracellular signal-regulated kinase
MEK: mitogen-activated protein kinase kinase
ES: electrical stimulation
MWCNT: multiwall carbon nanotubes
DCM: methylene dichloride
APFM: aligned PLLA fiber mat
HAPFM: highly aligned PLLA fiber mat
CNTs: carbon nanotubes
SEM: scanning electron microscope
FFT: fast Fourier transform spectrogram
PDMS: polydimethylsiloxane
SFI: sciatic functional index
CMAP: compound muscle action potential
NCV: nerve conduction velocity
LBF: Luxol Fast Blue
TB: Toluidine Blue
NGF: nerve growth factor
PNS: peripheral nervous system
FDA: Food and Drug Administration
BDNF: brain-derived neurotrophic factor
cAMP: cyclic adenosine monophosphate
CREB: cAMP response element binding protein
p75-NgR: p75-Nogo receptor
VEGF: vascular endothelial growth factor
PTN: pluripotent growth factor
TRK: tyrosine receptor kinase.

## References

[B1] Ahadian S. Ostrovidov S. Hosseini V. Kaji H. Ramalingam M. Bae H. (2013). Electrical stimulation as a biomimicry tool for regulating muscle cell behavior. *Organogenesis* 9 87–92. 10.4161/org.25121 23823664 PMC3812291

[B2] Alike Y. Yushan M. Keremu A. Abulaiti A. Liu Z. H. Fu W. (2019). Application of custom anatomy-based nerve conduits on rabbit sciatic nerve defects: *in vitro* and *in vivo* evaluations. *Neural Regen. Res.* 14 2173–2182. 10.4103/1673-5374.262601 31397357 PMC6788245

[B3] Al-Majed A. A. Neumann C. M. Brushart T. M. Gordon T. (2000). Brief electrical stimulation promotes the speed and accuracy of motor axonal regeneration. *J. Neurosci.* 20 2602–2608. 10.1523/jneurosci.20-07-02602.2000 10729340 PMC6772244

[B4] Amniattalab A. Mohammadi R. (2017). Functional, histopathological and immunohistichemical assessments of cyclosporine A on sciatic nerve regeneration using allografts: a rat sciatic nerve model. *Bull. Emerg. Trauma* 5 152–159. 28795058 PMC5547201

[B5] Cargnello M. Roux P. P. (2011). Activation and function of the MAPKs and their substrates, the MAPK-activated protein kinases. *Microbiol. Mol. Biol. Rev.* 75 50–83. 10.1128/MMBR.00031-10 21372320 PMC3063353

[B6] Chang W. Shah M. B. Lee P. Yu X. (2018). Tissue-engineered spiral nerve guidance conduit for peripheral nerve regeneration. *Acta Biomater.* 73 302–311. 10.1016/j.actbio.2018.04.046 29702292

[B7] Cheah M. Fawcett J. W. Haenzi B. (2017). Differential regenerative ability of sensory and motor neurons. *Neurosci. Lett.* 652 35–40. 10.1016/j.neulet.2016.11.004 27818349

[B8] Chi N. Wang R. (2018). Electrospun protein-CNT composite fibers and the application in fibroblast stimulation. *Biochem. Biophys. Res. Commun.* 504 211–217. 10.1016/j.bbrc.2018.08.157 30172370

[B9] Cho Y. I. Choi J. S. Jeong S. Y. Yoo H. S. (2010). Nerve growth factor (NGF)-conjugated electrospun nanostructures with topographical cues for neuronal differentiation of mesenchymal stem cells. *Acta Biomater.* 6 4725–4733. 10.1016/j.actbio.2010.06.019 20601229

[B10] Cho Y. Borgens R. B. (2010). The effect of an electrically conductive carbon nanotube/collagen composite on neurite outgrowth of PC12 cells. *J. Biomed. Mater. Res. A* 95 510–517. 10.1002/jbm.a.32841 20665676

[B11] Chomiak T. Hu B. (2009). What is the optimal value of the g-ratio for myelinated fibers in the rat CNS? A theoretical approach. *PLoS One* 4:e7754. 10.1371/journal.pone.0007754 19915661 PMC2771903

[B12] Chrzaszcz P. Derbisz K. Suszynski K. Miodonski J. Trybulski R. Lewin-Kowalik J. (2018). Application of peripheral nerve conduits in clinical practice: a literature review. *Neurol. Neurochir. Pol.* 52 427–435. 10.1016/j.pjnns.2018.06.003 30025722

[B13] Corletto A. Shapter J. G. (2020). Nanoscale patterning of carbon nanotubes: techniques, applications, and future. *Adv. Sci. (Weinh)* 8:2001778. 10.1002/advs.202001778 33437571 PMC7788638

[B14] Dechiara T. M. Bowen D. C. Valenzuela D. M. Simmons M. V. Poueymirou W. T. Thomas S. (1996). The receptor tyrosine kinase MuSK is required for neuromuscular junction formation *in vivo*. *Cell* 85:501. 10.1016/s0092-8674(00)81251-9 8653786

[B15] Desimone D. Li W. Roether J. A. Schubert D. W. Crovace M. C. Rodrigues A. C. (2013). Biosilicate((R))-gelatine bone scaffolds by the foam replica technique: development and characterization. *Sci. Technol. Adv. Mater.* 14:045008. 10.1088/1468-6996/14/4/045008 27877601 PMC5090327

[B16] Evans G. R. D. Brandt K. Katz S. Chauvin P. Otto L. Bogle M. (2002). Bioactive poly(l-lactic acid) conduits seeded with Schwann cells for peripheral nerve regeneration. *Biomaterials* 23 841–848. 10.1016/s0142-9612(01)00190-9 11774850

[B17] Gao Q. Gu H. Zhao P. Zhang C. Cao M. Fu J. (2018). Fabrication of electrospun nanofibrous scaffolds with 3D controllable geometric shapes. *Mater. Design* 157 159–169. 10.1016/j.matdes.2018.07.042

[B18] Geremia N. M. Gordon T. Brushart T. M. Al-Majed A. A. Verge V. M. (2007). Electrical stimulation promotes sensory neuron regeneration and growth-associated gene expression. *Exp. Neurol.* 205 347–359. 10.1016/j.expneurol.2007.01.040 17428474

[B19] Gordon T. (2009). The role of neurotrophic factors in nerve regeneration. *Neurosurg. Focus* 26:E3. 10.3171/FOC.2009.26.2.E3 19228105

[B20] Gordon T. (2016). Electrical stimulation to enhance axon regeneration after peripheral nerve injuries in animal models and humans. *Neurotherapeutics* 13 295–310. 10.1007/s13311-015-0415-1 26754579 PMC4824030

[B21] Gordon T. Amirjani N. Edwards D. C. Chan K. M. (2010). Brief post-surgical electrical stimulation accelerates axon regeneration and muscle reinnervation without affecting the functional measures in carpal tunnel syndrome patients. *Exp. Neurol.* 223 192–202. 10.1016/j.expneurol.2009.09.020 19800329

[B22] Gordon T. Borschel G. H. (2017). The use of the rat as a model for studying peripheral nerve regeneration and sprouting after complete and partial nerve injuries. *Exp. Neurol.* 287(Pt. 3) 331–347. 10.1016/j.expneurol.2016.01.014 26795087

[B23] Gordon T. Tyreman N. Raji M. A. (2011). The basis for diminished functional recovery after delayed peripheral nerve repair. *J. Neurosci.* 31 5325–5334. 10.1523/JNEUROSCI.6156-10.2011 21471367 PMC6622714

[B24] Gribble K. D. Walker L. J. Saint-Amant L. Kuwada J. Y. Granato M. (2018). The synaptic receptor Lrp4 promotes peripheral nerve regeneration. *Nat. Commun.* 9:2389. 10.1038/s41467-018-04806-4 29921864 PMC6008306

[B25] Haastert-Talini K. Schmitte R. Korte N. Klode D. Ratzka A. Grothe C. (2011). Electrical stimulation accelerates axonal and functional peripheral nerve regeneration across long gaps. *J. Neurotrauma* 28 661–674. 10.1089/neu.2010.1637 21265597

[B26] Hu X. Huang J. Ye Z. Xia L. Li M. Lv B. (2009). A novel scaffold with longitudinally oriented microchannels promotes peripheral nerve regeneration. *Tissue Eng. Part A* 15 3297–3308. 10.1089/ten.TEA.2009.0017 19382873

[B27] Hurtado A. Cregg J. M. Wang H. B. Wendell D. F. Oudega M. Gilbert R. J. (2011). Robust CNS regeneration after complete spinal cord transection using aligned poly-L-lactic acid microfibers. *Biomaterials* 32 6068–6079. 10.1016/j.biomaterials.2011.05.006 21636129 PMC4163047

[B28] Jahromi H. K. Farzin A. Hasanzadeh E. Barough S. E. Mahmoodi N. Najafabadi M. R. H. (2020). Enhanced sciatic nerve regeneration by poly-L-lactic acid/multi-wall carbon nanotube neural guidance conduit containing Schwann cells and curcumin encapsulated chitosan nanoparticles in rat. *Mater. Sci. Eng. C Mater. Biol. Appl.* 109:110564. 10.1016/j.msec.2019.110564 32228906

[B29] Ji Y. Shen M. Wang X. Zhang S. Yu S. Chen G. (2012). Comparative proteomic analysis of primary Schwann cells and a spontaneously immortalized Schwann cell line RSC 96: a comprehensive overview with a focus on cell adhesion and migration related proteins. *J. Proteome Res.* 11 3186–3198. 10.1021/pr201221u 22519560

[B30] Kaplan B. Merdler U. Szklanny A. A. Redenski I. Guo S. Bar-Mucha Z. (2020). Rapid prototyping fabrication of soft and oriented polyester scaffolds for axonal guidance. *Biomaterials* 251:120062. 10.1016/j.biomaterials.2020.120062 32388032

[B31] Kawamura K. Kano Y. (2019). Electrical stimulation induces neurite outgrowth in PC12m3 cells via the p38 mitogen-activated protein kinase pathway. *Neurosci. Lett.* 698 81–84. 10.1016/j.neulet.2019.01.015 30634009

[B32] Kornfeld T. Vogt P. M. Radtke C. (2019). Nerve grafting for peripheral nerve injuries with extended defect sizes. *Wien. Med. Wochenschr.* 169 240–251. 10.1007/s10354-018-0675-6 30547373 PMC6538587

[B33] Lanone S. Andujar P. Kermanizadeh A. Boczkowski J. (2013). Determinants of carbon nanotube toxicity. *Adv. Drug Deliv. Rev.* 65 2063–2069. 10.1016/j.addr.2013.07.019 23928473

[B34] Lin C. Liu C. Zhang L. Huang Z. Zhao P. Chen R. (2018). Interaction of iPSC-derived neural stem cells on poly(L-lactic acid) nanofibrous scaffolds for possible use in neural tissue engineering. *Int. J. Mol. Med.* 41 697–708. 10.3892/ijmm.2017.3299 29207038 PMC5752187

[B35] Liu Z. Yushan M. Alike Y. Liu Y. Wu S. Ma C. (2020). Preparation of multiwall carbon nanotubes embedded electroconductive multi-microchannel scaffolds for neuron growth under electrical stimulation. *Biomed Res. Int.* 2020:4794982. 10.1155/2020/4794982 32337253 PMC7153003

[B36] Love M. R. Palee S. Chattipakorn S. C. Chattipakorn N. (2018). Effects of electrical stimulation on cell proliferation and apoptosis. *J. Cell. Physiol.* 233 1860–1876. 10.1002/jcp.25975 28452188

[B37] MacDonald R. A. Laurenzi B. F. Viswanathan G. Ajayan P. M. Stegemann J. P. (2005). Collagen-carbon nanotube composite materials as scaffolds in tissue engineering. *J. Biomed. Mater. Res. A* 74 489–496. 10.1002/jbm.a.30386 15973695

[B38] Magnaghi V. Conte V. Procacci P. Pivato G. Cortese P. Cavalli E. (2011). Biological performance of a novel biodegradable polyamidoamine hydrogel as guide for peripheral nerve regeneration. *J. Biomed. Mater. Res. A* 98 19–30. 10.1002/jbm.a.33091 21509933

[B39] Mahairaki V. Lim S. H. Christopherson G. T. Xu L. Nasonkin I. Yu C. (2011). Nanofiber matrices promote the neuronal differentiation of human embryonic stem cell-derived neural precursors *in vitro*. *Tissue Eng. Part A* 17 855–863. 10.1089/ten.TEA.2010.0377 20973749 PMC3043983

[B40] McGregor C. E. English A. W. (2018). The role of BDNF in peripheral nerve regeneration: activity-dependent treatments and Val66Met. *Front. Cell. Neurosci.* 12:522. 10.3389/fncel.2018.00522 30687012 PMC6336700

[B41] Menezes B. R. C. Rodrigues K. F. Fonseca B. Ribas R. G. Montanheiro T. Thim G. P. (2019). Recent advances in the use of carbon nanotubes as smart biomaterials. *J. Mater. Chem. B* 7 1343–1360. 10.1039/c8tb02419g 32255006

[B42] Modrak M. Talukder M. A. H. Gurgenashvili K. Noble M. Elfar J. C. (2020). Peripheral nerve injury and myelination: potential therapeutic strategies. *J. Neurosci. Res.* 98 780–795. 10.1002/jnr.24538 31608497 PMC7072007

[B43] Moroder P. Runge M. B. Wang H. Ruesink T. Lu L. Spinner R. J. (2011). Material properties and electrical stimulation regimens of polycaprolactone fumarate-polypyrrole scaffolds as potential conductive nerve conduits. *Acta Biomater.* 7 944–953. 10.1016/j.actbio.2010.10.013 20965280 PMC3031729

[B44] Morrill E. E. Tulepbergenov A. N. Stender C. J. Lamichhane R. Brown R. J. Lujan T. J. (2016). A validated software application to measure fiber organization in soft tissue. *Biomech. Model. Mechanobiol.* 15 1467–1478. 10.1007/s10237-016-0776-3 26946162 PMC5328598

[B45] Niu Y. Stadler F. J. Fu M. (2021). Biomimetic electrospun tubular PLLA/gelatin nanofiber scaffold promoting regeneration of sciatic nerve transection in SD rat. *Mater. Sci. Eng. C Mater. Biol. Appl.* 121:111858. 10.1016/j.msec.2020.111858 33579490

[B46] Qiu T. Yin Y. Li B. Xie L. Yan Q. Dai H. (2014). PDLLA/PRGD/beta-TCP conduits build the neurotrophin-rich microenvironment suppressing the oxidative stress and promoting the sciatic nerve regeneration. *J. Biomed. Mater. Res. A* 102 3734–3743. 10.1002/jbm.a.35078 24408878

[B47] Rao F. Wang Y. Zhang D. Lu C. Cao Z. Sui J. (2020). Aligned chitosan nanofiber hydrogel grafted with peptides mimicking bioactive brain-derived neurotrophic factor and vascular endothelial growth factor repair long-distance sciatic nerve defects in rats. *Theranostics* 10 1590–1603. 10.7150/thno.36272 32042324 PMC6993237

[B48] Raphey V. R. Henna T. K. Nivitha K. P. Mufeedha P. Sabu C. Pramod K. (2019). Advanced biomedical applications of carbon nanotube. *Mater. Sci. Eng. C Mater. Biol. Appl.* 100 616–630. 10.1016/j.msec.2019.03.043 30948098

[B49] Rbia N. Bulstra L. F. Saffari T. M. Hovius S. E. R. Shin A. Y. (2019). Collagen nerve conduits and processed nerve allografts for the reconstruction of digital nerve gaps: a single-institution case series and review of the literature. *World Neurosurg.* 127 e1176–e1184. 10.1016/j.wneu.2019.04.087 31003028

[B50] Romero M. I. Lin L. Lush M. E. Lei L. Parada L. F. Zhu Y. (2007). Deletion of Nf1 in neurons induces increased axon collateral branching after dorsal root injury. *J. Neurosci.* 27 2124–2134. 10.1523/JNEUROSCI.4363-06.2007 17314307 PMC6673560

[B51] Saleemi M. A. Hosseini Fouladi M. Yong P. V. C. Chinna K. Palanisamy N. K. Wong E. H. (2021). Toxicity of carbon nanotubes: molecular mechanisms, signaling cascades, and remedies in biomedical applications. *Chem. Res. Toxicol.* 34 24–46. 10.1021/acs.chemrestox.0c00172 33319996

[B52] Saudi A. Amini S. Amirpour N. Kazemi M. Zargar Kharazi A. Salehi H. (2019). Promoting neural cell proliferation and differentiation by incorporating lignin into electrospun poly(vinyl alcohol) and poly(Glycerol sebacate) fibers. *Mater. Sci. Eng. C Mater. Biol. Appl.* 104:110005. 10.1016/j.msec.2019.110005 31499996

[B53] Scholz T. Krichevsky A. Sumarto A. Jaffurs D. Wirth G. A. Paydar K. (2009). Peripheral nerve injuries: an international survey of current treatments and future perspectives. *J. Reconstr. Microsurg.* 25 339–344. 10.1055/s-0029-1215529 19301234

[B54] Sit S. T. Manser E. (2011). Rho GTPases and their role in organizing the actin cytoskeleton. *J. Cell Sci.* 124(Pt. 5) 679–683. 10.1242/jcs.064964 21321325

[B55] Theodore N. Hlubek R. Danielson J. Neff K. Vaickus L. Ulich T. R. (2016). First human implantation of a bioresorbable polymer scaffold for acute traumatic spinal cord injury: a clinical pilot study for safety and feasibility. *Neurosurgery* 79 E305–E312. 10.1227/NEU.0000000000001283 27309344

[B56] Thrivikraman G. Boda S. Basu B. (2018). Unraveling the mechanistic effects of electric field stimulation towards directing stem cell fate and function: a tissue engineering perspective. *Biomaterials* 150 60–86. 10.1016/j.biomaterials.2017.10.003 29032331

[B57] Varejão A. S. Meek M. F. Ferreira A. J. Patrício J. A. Cabrita A. M. (2001). Functional evaluation of peripheral nerve regeneration in the rat: walking track analysis. *J. Neurosci. Methods* 108 1–9. 10.1016/s0165-0270(01)00378-8 11459612

[B58] Wang H. B. Mullins M. E. Cregg J. M. McCarthy C. W. Gilbert R. J. (2010). Varying the diameter of aligned electrospun fibers alters neurite outgrowth and Schwann cell migration. *Acta Biomater.* 6 2970–2978. 10.1016/j.actbio.2010.02.020 20167292

[B59] Wieringa P. A. Goncalves de Pinho A. R. Micera S. van Wezel R. J. A. Moroni L. (2018). Biomimetic architectures for peripheral nerve repair: a review of biofabrication strategies. *Adv. Healthc. Mater.* 7:e1701164. 10.1002/adhm.201701164 29349931

[B60] Wu R. Wang L. Chen F. Huang Y. Shi J. Zhu X. (2016). Evaluation of artificial nerve conduit and autografts in peripheral nerve repair in the rat model of sciatic nerve injury. *Neurol. Res.* 38 461–466. 10.1080/01616412.2016.1181346 27146214

[B61] Xie J. MacEwan M. R. Schwartz A. G. Xia Y. (2010). Electrospun nanofibers for neural tissue engineering. *Nanoscale* 2 35–44. 10.1039/b9nr00243j 20648362

[B62] Xue C. Zhu H. Tan D. Ren H. Gu X. Zhao Y. (2018). Electrospun silk fibroin-based neural scaffold for bridging a long sciatic nerve gap in dogs. *J. Tissue Eng. Regen. Med.* 12 e1143–e1153. 10.1002/term.2449 28485084

[B63] Yang F. Murugan R. Wang S. Ramakrishna S. (2005). Electrospinning of nano/micro scale poly(L-lactic acid) aligned fibers and their potential in neural tissue engineering. *Biomaterials* 26 2603–2610. 10.1016/j.biomaterials.2004.06.051 15585263

[B64] Yu K. Zhang C. Wang Y. Zhang P. Zhang D. Zhang H. (2009). The protective effects of small gap sleeve in bridging peripheral nerve mutilation. *Artif. Cells Blood Substit. Immobil. Biotechnol.* 37 257–264. 10.3109/10731190903360810 19900064

[B65] Yu Y. Lu X. Ding F. (2015). Influence of poly(L-Lactic Acid) aligned nanofibers on PC12 differentiation. *J. Biomed. Nanotechnol.* 11 816–827. 10.1166/jbn.2015.1973 26349394

[B66] Zhang Q. Tong Z. Chen F. Wang X. Ren M. Zhao Y. (2020). Aligned soy protein isolate-modified poly(L-lactic acid) nanofibrous conduits enhanced peripheral nerve regeneration. *J. Neural Eng.* 17:036003. 10.1088/1741-2552/ab8d81 32340001

[B67] Zhao Y. Liang Y. Ding S. Zhang K. Mao H. Q. Yang Y. (2020). Application of conductive PPy/SF composite scaffold and electrical stimulation for neural tissue engineering. *Biomaterials* 255:120164. 10.1016/j.biomaterials.2020.120164 32554132

[B68] Zizzo J. (2021). Muscle atrophy classification: the need for a pathway-driven approach. *Curr. Pharm. Des.* 27 3012–3019. 10.2174/1381612824666210316102413 33726645

[B69] Zuo K. J. Gordon T. Chan K. M. Borschel G. H. (2020). Electrical stimulation to enhance peripheral nerve regeneration: update in molecular investigations and clinical translation. *Exp. Neurol.* 332:113397. 10.1016/j.expneurol.2020.113397 32628968

