# Supplementary material for: Enhanced Nerve Regeneration by Bionic Conductive Nerve Scaffold Under Electrical Stimulation
Source: Front Neurosci. 2022 Apr 27;16:810676. doi: 10.3389/fnins.2022.810676 (PMC9091912; doi:10.3389/fnins.2022.810676)
Supplement: Supplementary file 1 [file Data_Sheet_1.docx]

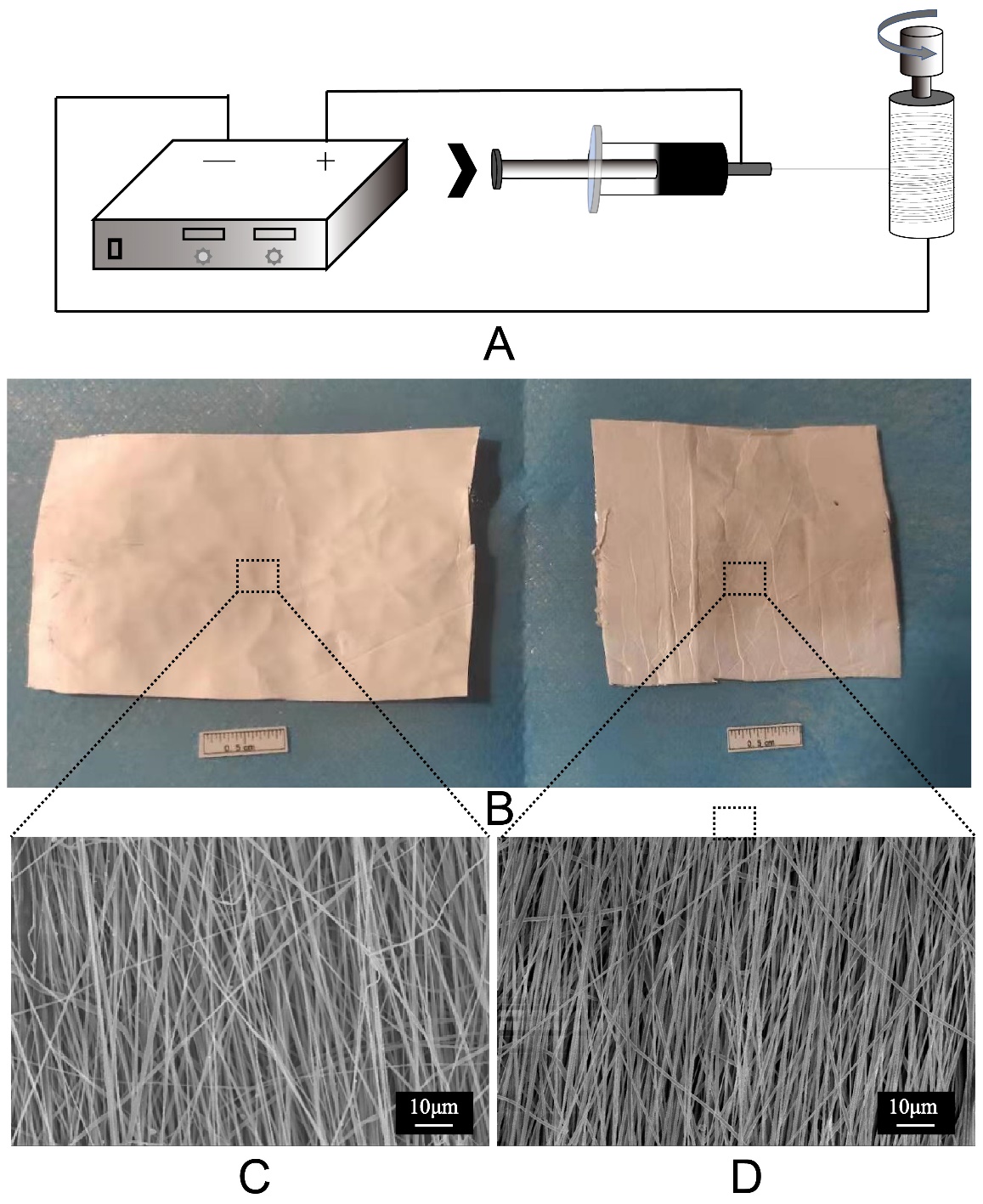


**S 1** Preparation of PLLA fiber mats. A: Schematic diagram of preparation of PLLA fiber mats by electrospinning. B: The appearances of aligned PLLA fiber mat (left) and highly aligned PLLA fiber mat. C: The structure of APFM observed by SEM. D: The structure of HAPFM observed by SEM.


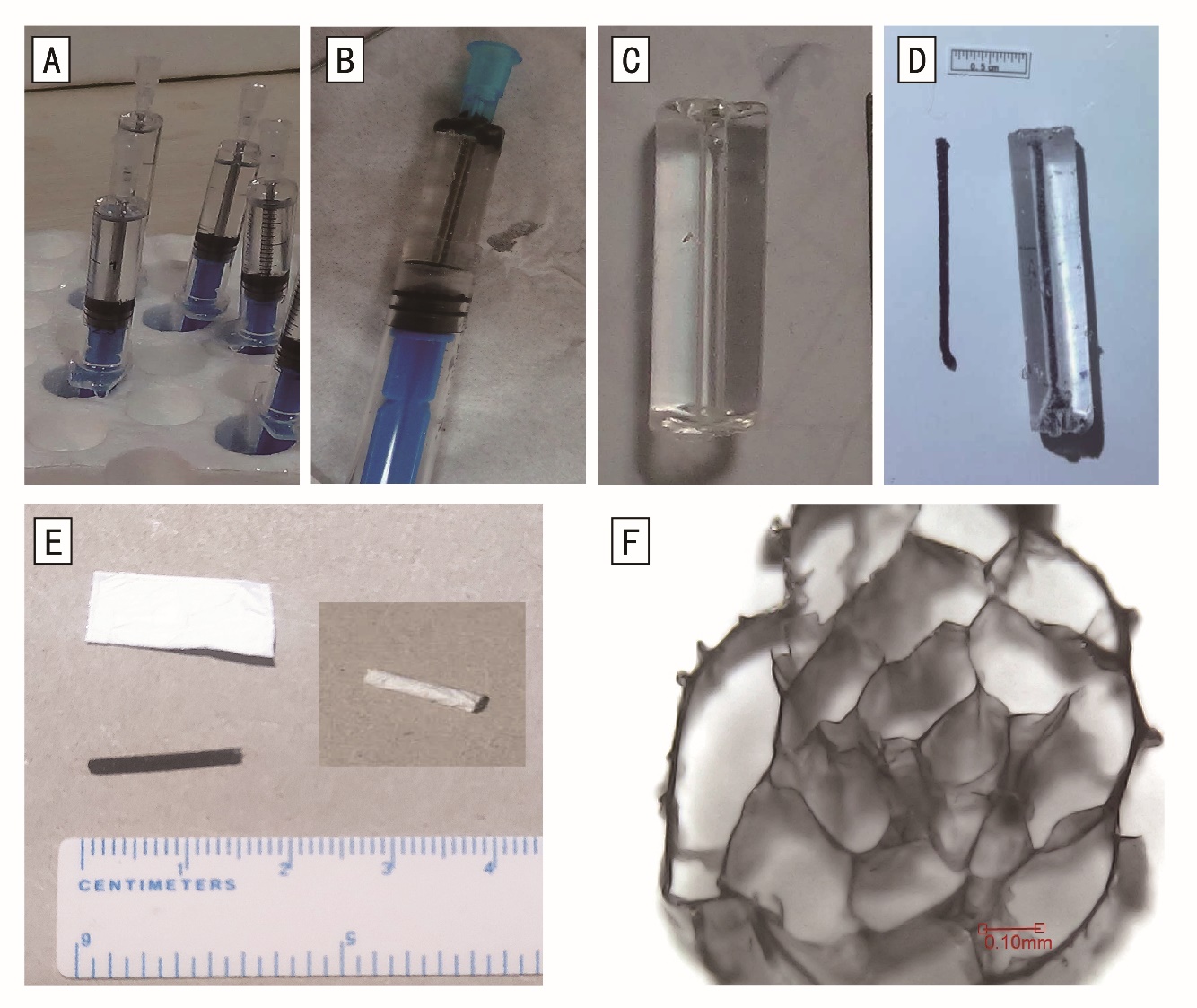


**S2** Pictures of the mold and the nerve scaffolds. A: The solidifying PDMS mold; B: The solidified PDMS mold; C: PDMS mold with inner diameter of 1.5mm; D: Customized conductive nerve scaffold with multi-microchannels prepared by molds; E: A bionic conductive nerve scaffold composed of a HAPFM and a conductive nerve scaffold. F: The cross section of nerve scaffold with multi-microchannels.


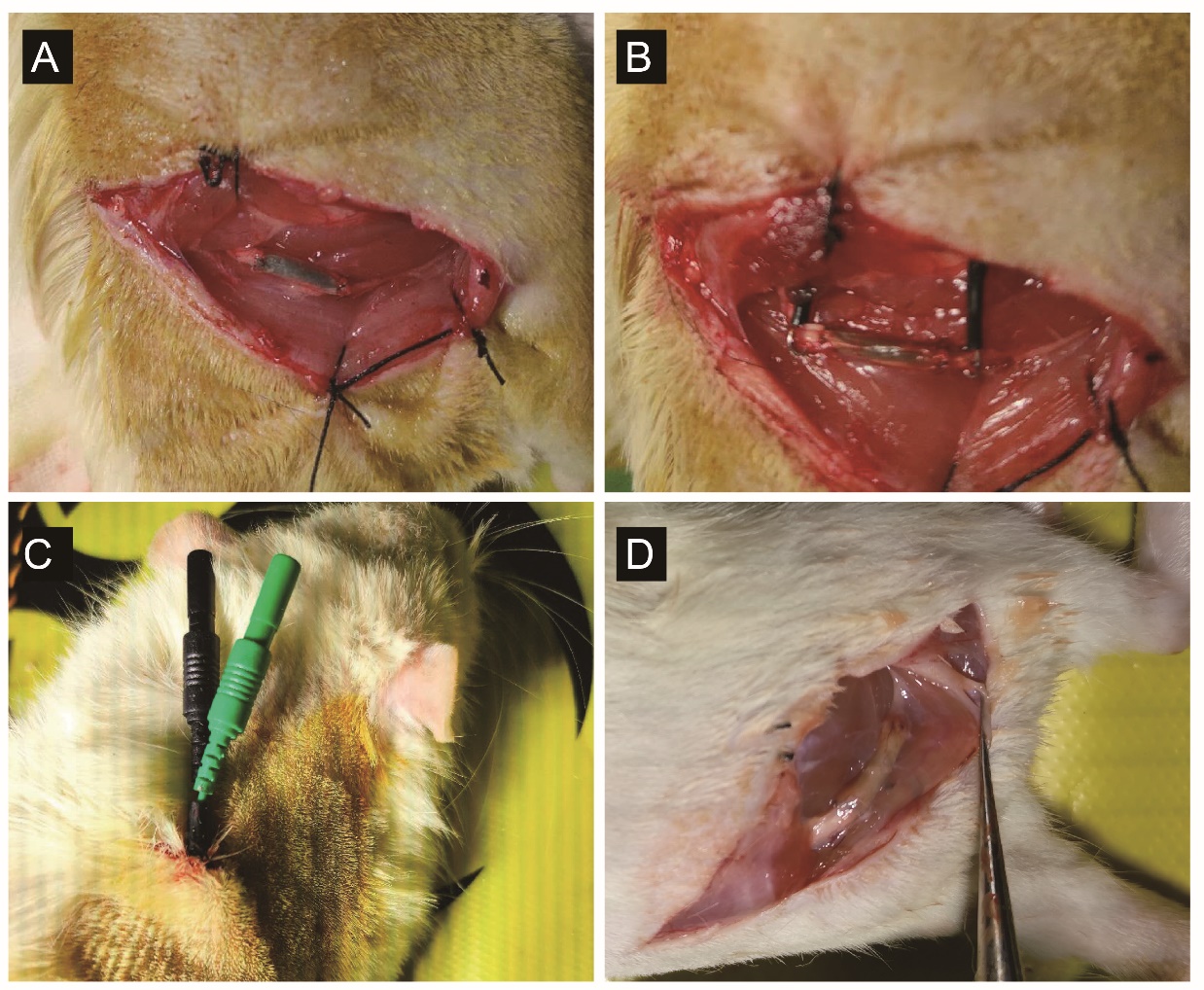


**S3** Animal experiment procedure. A: The biomimetic conductive artificial nerve scaffold was implanted into the defect of rat sciatic nerve; B: Electrodes implanted; C: Electrode guide wires fixed; D: General image at 12W after implantation.
